# Propyl Gallate Exerts an Antimigration Effect on Temozolomide-Treated Malignant Glioma Cells through Inhibition of ROS and the NF-*κ*B Pathway

**DOI:** 10.1155/2017/9489383

**Published:** 2017-09-14

**Authors:** Jen-Tsung Yang, I-Neng Lee, Fung-Jou Lu, Chiu-Yen Chung, Ming-Hsueh Lee, Yu-Ching Cheng, Kuo-Tai Chen, Ching-Hsein Chen

**Affiliations:** ^1^Department of Neurosurgery, Chang Gung Memorial Hospital, Chiayi 61363, Taiwan; ^2^College of Medicine, Chang Gung University, Tao-Yuan 33302, Taiwan; ^3^Department of Medical Research, Chang Gung Memorial Hospital, Chiayi 61363, Taiwan; ^4^Institute of Medicine, Chung Shan Medical University, Taichung, Taiwan; ^5^Department of Microbiology, Immunology and Biopharmaceuticals, College of Life Sciences, National Chiayi University, Chiayi City 60004, Taiwan

## Abstract

In this study, we demonstrated that temozolomide (TMZ) and propyl gallate (PG) combination enhanced the inhibition of migration in human U87MG glioma cells. PG inhibited the TMZ-induced reactive oxygen species (ROS) generation. The mitochondrial complex III and NADPH oxidase are two critical sites that can be considered to regulate antimigration in TMZ-treated U87MG cells. PG can enhance the antimigration effect of TMZ through suppression of metalloproteinase-2 and metalloproteinase-9 activities, ROS generation, and the NF-*κ*B pathway and possibly provide a novel prospective strategy for treating malignant glioma.

## 1. Introduction

Malignant gliomas, the most common primary brain tumor with an annual incidence of about 5.26 cases per 100,000 people, have high growth rates and necrotic characteristics [1]. Glioblastoma multiforme (GBM), a type of malignant glioma, is one of the most difficult cancers to treat, and median survival is about 14.6 months in spite of aggressive treatment with surgery, radiotherapy, chemotherapy, and immunotherapy [2]. Temozolomide (TMZ) is an oral alkylating and well-tolerated chemotherapeutic drug and has been approved for the treatment of malignant glioma clinically for a long time. However, O^6^-methylguanine-DNA methyltransferase gene (MGMT) promoter methylation status influences the efficacy of the drug, and therapeutic outcomes are still unsatisfactory [3]. In addition, GBM is highly neovascularizated with the appearance of angiogenesis and vasculogenesis. The modification of preexisting blood vessels combined with antiangiogenesis drugs for blocking new vessel formation may possibly improve the therapeutic outcome [4]. Bevacizumab, a vascular endothelial cell proliferation inhibitor, is currently an alternative treatment choice for GBM but fails to extend overall survival time [5, 6]. In experimental studies, drugs such as betulinic acid, lonidamine, and CD437, which target the mitochondrial pore [7–9], and crizotinib, which inhibit mesenchymal-epithelial transition (MET) expression [10], possibly provide a strategy for GBM therapy. Our previous study also demonstrated that valproic acid could enhance the apoptotic effect of TMZ through a redox regulation mechanism [11].

Propyl gallate (PG), a polyphenolic compound family that is synthesized by the condensation of propanol and gallic acid, has an antiproliferation effect on tumor cells [12]. In one study, PG could modulate heme oxygenase-1 (HO-1) activation and decrease lung cancer cell survival [13]. PG also induces apoptosis in human leukemia cells [14] and HeLa cells [15] by increasing reactive oxygen species (ROS) levels and glutathione (GSH) depletion. In brain ischemia, PG inhibits the activity of NF-*κ*B, reduces COX-2 and TNF-alpha G expression, and decreases ischemic-reperfusion injury [16]. However, PG effects on brain glioma cells are still questionable and not well investigated. In this study, we examined whether PG can potentiate the antimigration effects of TMZ on malignant glioma cells and elucidate the possible molecular mechanisms.

## 2. Materials and Methods

### 2.1. Cell Lines, Reagents, and Chemicals

U87MG (a human primary glioblastoma cell line) was obtained from the Bioresource Collection and Research Center (Food Industry Research and Development Institute, Hsinchu, Taiwan). HBVP (a human brain vascular pericyte cell line), HA (a human astrocyte cell line), and HBMEC (a human brain microvascular endothelial cell line) were purchased from ScienCell Research Laboratories Inc. (Carlsbad, CA, USA). Gibco™ Eagle's minimum essential medium (MEM) and fetal bovine serum (FBS) were purchased from Thermo Fisher Scientific (Waltham, MA, USA). Pericyte medium (PM), astrocyte medium (AM), and endothelial cell medium (ECM) were obtained from ScienCell Research Laboratories Inc. (Carlsbad, CA, USA). Primary antibodies against I*_κ_*B kinase (IKK), I*_κ_*B, p65, p-IKK, p-I*_κ_*B, p-p65, lamin B, and *β*-actin were obtained from Santa Cruz Biotechnology Inc. (Dallas, TX, USA). The Bradford protein assay reagent for protein concentration determination was purchased from Bio-Rad Laboratories Inc. (Hercules, CA, USA). PG, propidium iodide (PI), TMZ, 2′,7′-dichlorodihydrofluorescein diacetate (DCFDA), dimethyl sulfoxide (DMSO), Trypan blue solution, crystal violet, and other chemicals were purchased from Sigma-Aldrich Corp. (St. Louis, MO, USA).

### 2.2. Cell Culture and Drug Treatment

U87MG cells were cultured in MEM containing 10% FBS, 2 mM L-glutamine, 100 units/mL penicillin G, and 100 *μ*g/mL streptomycin and placed in a 5% CO_2_/95% incubator at 37°C. HBVP cells, HA cells, and HBMEC cells were cultured in PM, AM, and ECM, respectively, and placed in a 5% CO_2_/95% incubator at 37°C. PM consists of 500 mL of basal medium, 10 mL of FBS, 5 mL of pericyte growth supplement, and 5 mL of penicillin/streptomycin solution. AM consists of 500 mL of basal medium, 10 mL of FBS, 5 mL of astrocyte growth supplement, and 5 mL of penicillin/streptomycin solution. ECM consists of 500 mL of basal medium, 25 mL of FBS, 5 mL of endothelial cell growth supplement, and 5 mL of penicillin/streptomycin solution. The stock solutions of PG or TMZ were prepared in DMSO, and all treated concentrations were diluted in culture medium. The concentration of DMSO should not be exceeding from 0.05%.

### 2.3. Cytotoxicity Assay

Cytotoxicity was evaluated using a cell proliferation kit (Biological Industries; Kibbutz Beit Haemek, Israel). A total of 1 × 10^4^ cells per well were seeded in a flat bottom 96-well plate before treatment. After treatment, cells in a 100 *μ*L medium were incubated with 50 *μ*L XTT reagent for 2 h, and the absorbance of the sample (450 nm) was measured against a background control medium as a blank. The nonspecific absorbance (690 nm) was measured and subtracted from the 450 nm measurement in an EnSpire® multimode plate reader (PerkinElmer; Billerica, MA, USA).

### 2.4. Transwell Migration Assay

Transwell migration assay was carried out using 24-well Millicell® hanging cell culture inserts with a polyethylene terephthalate (PET) membrane pore size of 8 *μ*m (EMD Millipore; Billerica, MA, USA). Cells (1 × 10^4^ cells/well) were seeded in a serum-free medium to triplicate wells of cell culture inserts, and a complete medium containing 10% FBS was added to the lower chamber. After 48 h, the interior of the inserts was swabbed to remove nonmigratory cells, and those migratory cells that passed through the PET membrane were fixed and stained using crystal violet solution. At least three individual fields per insert were counted for cell number using a phase contrast microscope. Then, each insert was transferred to an empty well containing 200 *μ*L extraction solution (33% acetic acid) to lyse the cells, and 100 *μ*L from each sample was transferred and measured at 560 nm using an EnSpire multimode plate reader (PerkinElmer).

### 2.5. Analysis of the Activities of Matrix Metalloproteinase- (MMP-) 2 and MMP-9 in Human U87MG Cells

U87MG cells (2 × 10^6^) were cultured in 60 mm culture dishes for 48 h. The cells were then exposed to various concentrations of TMZ alone, PG alone, or combined PG and TMZ for appropriate times. After treatment, the cultured media were collected and then centrifuged at 12,000 ×g for 10 min at 4°C to obtain the supernatant. The protein concentrations of supernatant were measured with a Bio-Rad protein assay reagent (Hercules, CA, USA). The enzyme activities of MMP-2 and MMP-9 were evaluated by zymography. Fifty micrograms of the supernatant protein were subjected to electrophoresis in a 10% SDS-PAGE gel copolymerized with gelatin (1 mg/mL). After electrophoresis, the gels were then washed in distilled water for 5 min and then incubated for 1 h in 2.5% Triton-X solution in order to remove the SDS [17]. It would restore the protein 3D structure and functionality. The gelatinolytic activities of MMP-2 and MMP-9 were evaluated as transparent bands against a background of coomassie brilliant blue-stained gelatin and quantified using Bio-Rad Quantity One® 1-D analysis software (Hercules, CA, USA).

### 2.6. Intracellular ROS Measurement

The production of intracellular ROS was detected by flow cytometry using 2′,7′-dichlorofluorescin diacetate (DCFDA). After drug treatment, the cells were stained with 20 *μ*M DCFDA for 30 min at 37°C and then washed with 1X PBS twice to remove the DCFDA. All cells were trypsinized to obtain a single-cell suspension. Intracellular ROS levels, which were indicated by the fluorescence of dichlorofluorescein (DCF), were measured through an excitation/emission = 485 nm/535 nm using a BD FACSCanto™ II flow cytometer (San Jose, CA, USA). Ten thousand cells were collected and analyzed per experimental conditions using mean fluorescent intensity.

### 2.7. Preparation of Nuclear Protein Extracts from U87MG Cells

After treatment, cells were washed with cold 1X PBS and suspended in 500 *μ*L of hypotonic lysis buffer (10 mM HEPES, pH 7.9, 1.5 mM MgCl_2_, and 10 mM KCl) containing protease inhibitors for 30 min. Then, 30 *μ*L of 10% NP-40 was added to the swollen cells in the lysis buffer and vortexed vigorously for 10 seconds. The homogenate was centrifuged immediately for 30 seconds at 11,000 ×g, and the supernatant containing the cytoplasmic extracts was stored at −80°C for further analysis. The nuclear pellet was resuspended in ~70 *μ*L of ice-cold nuclear extraction buffer (20 mM HEPES, pH 7.9, 1.5 mM MgCl_2_, 0.42 M NaCl, 0.2 mM EDTA, and 25% (v/v) glycerol). After 30 min of intermittent mixing, the extract was centrifuged at 20,000 ×g for 5 min, and the supernatants containing the nuclear extracts were secured. Nuclear p65 expression was evaluated by Western blotting.

### 2.8. Western Blot Analysis

For crude total protein extraction, cells were lysed in 1X buffer (25 mM Tris•HCl pH 7.6, 150 mM NaCl, 1% NP-40, 1% sodium deoxycholate, and 0.1% SDS) for 10 min and centrifuged at 12,000 ×g for 10 min at 4°C to obtain the soluble proteins. Twenty-five to fifty micrograms of protein were separated on a 12% SDS-polyacrylamide gel. After electrophoresis, the proteins were transferred onto a polyvinylidene fluoride (PVDF) membrane. The membranes were blocked with 5% nonfat milk, incubated with various primary antibodies overnight, and then washed with 1X PBST solution (0.05% Tween 20 in 1X PBS). After washing, the appropriate secondary antibodies, which were each labeled with horseradish peroxidase, were added to the membrane for 1 h and then washed with 1X PBST solution. The antigen-antibody complexes were detected using Amersham™ ECL™ Prime Western Blotting Detection Reagent (GE Healthcare Life Sciences; Uppsala, Sweden). Autoradiographic signals were detected by an X-ray film (Roche Applied Science, Mannheim, Germany). The signal intensity was quantitated by GeneTools analysis software (SYNGEN, Cambridge, UK).

### 2.9. Statistical Analysis

Data are presented as the mean ± standard deviation from at least three independent experiments and were analyzed using Student's *t*-tests. A *P* value of less than 0.05 was considered statistically significant.

## 3. Results

### 3.1. The Effect of Combined TMZ and PG on Cell Viability

**Figure 1:**
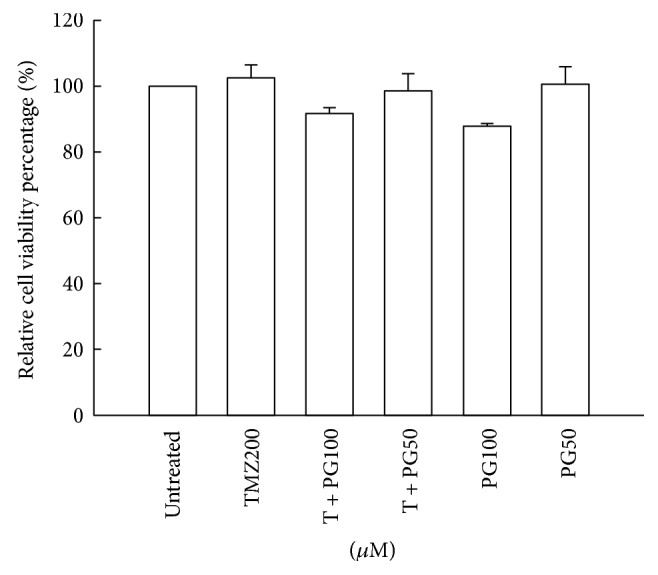
Evaluation of cell viability with TMZ, PG, or TMZ/PG treatment. U87MG cells (1 × 10^4^) were seeded in each well of a 96-well cultured plate for 24 h and then treated with TMZ (200 *μ*M), PG (50 or 100 *μ*M), or their combination for 48 h. After treatment, cell viability was determined by an XTT-based assay. The values are represented as mean ± standard deviation (*n* = 5–8) of individual experiments.

To exclude any confounding factors in the determination of migration inhibition by cell death, we first evaluated the effect of drug concentration on cell viability in U87MG cells. The U87MG cells maintained a cell viability of at least 85% when treated with TMZ (200 *μ*M) alone, PG alone (50 and 100 *μ*M), and combined TMZ and PG after 48 h (Figure 1). Concentrations of 50 and 100 *μ*M for PG and 200 *μ*M for TMZ were used in further studies.

### 3.2. PG Enhanced the Antimigration Effect of TMZ

**Figure 2:**
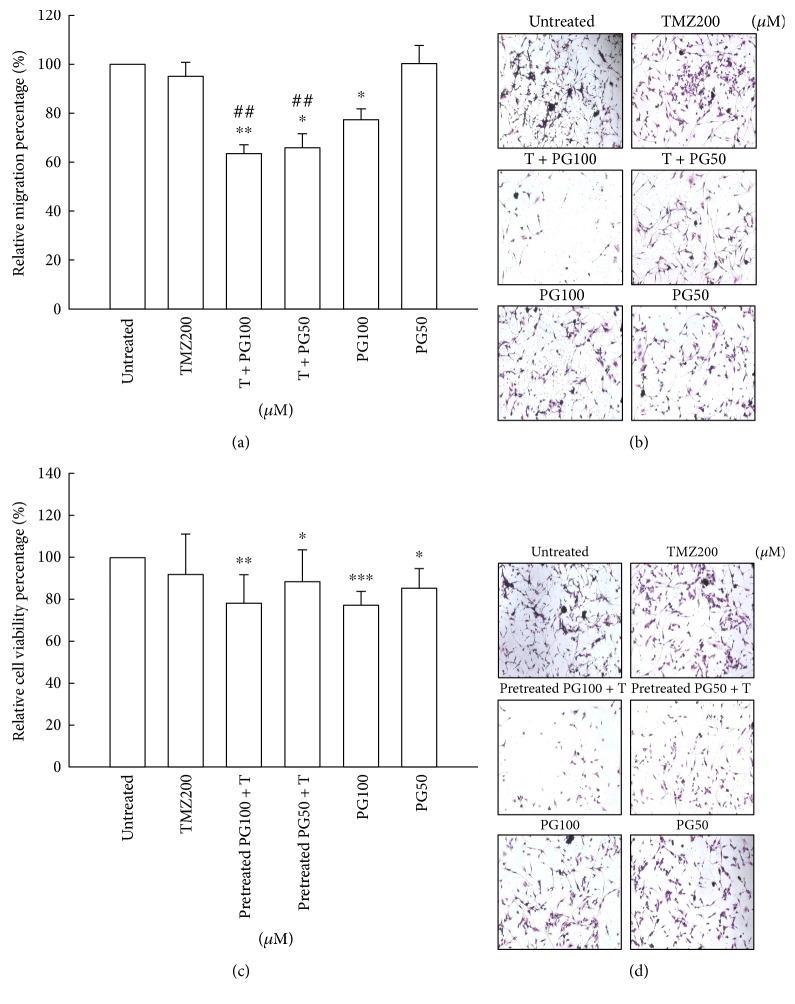
Evaluation of migration with TMZ, PG, or TMZ/PG treatment. (a, b) U87MG cells (1 × 10^4^) were plated in 24-well Millicell hanging cell culture inserts with an 8 *μ*m pore size membrane for 24 h and then treated with TMZ (200 *μ*M), PG (50 or 100 *μ*M), and their combination for 48 h. (c, d) U87MG cells (1 × 10^4^) were plated in 24-well Millicell hanging cell culture inserts with an 8 *μ*m pore size membrane for 24 h and then treated with TMZ (200 *μ*M) or PG (50 or 100 *μ*M) for 48 h or pretreated with PG (50 or 100 *μ*M) for 6 h and then treated with (200 *μ*M) for 48 h. (a, c) Transwell migration assay was carried out. The migrated cells were stained with crystal violet. The dye was eluted with 33% acetic acid, and crystal violet absorbance was measured at 570 nm using a microplate reader. The values are presented as mean ± standard deviation (*n* = 5–8) of individual experiments. Significant differences for the untreated group and TMZ group were ^∗^*P* < 0.05, ^∗∗^*P* < 0.01, ^∗∗∗^*P* < 0.001, and ^##^*P* < 0.01. (b, d) Random fields from each of the triplicate migration assays were counted using phase contrast microscopy (magnification 200x).

To determine whether PG has a potential role, either alone or in combination with TMZ, in the inhibition of migration in the treatment of glioma cells, the transwell migration assay and crystal violet staining method were used. There were no significant difference on antimigration in TMZ (200 *μ*M) alone or PG (50 *μ*M) alone compared with untreated cells (Figures 2(a) and 2(b)). PG (100 *μ*M) alone produced a significant effect on antimigration. The antimigration effect was further increased when PG (50 *μ*M) and TMZ (200 *μ*M) or PG (100 *μ*M) and TMZ (200 *μ*M) in U87MG cells are combined. To evaluate the effect of PG pretreated TMZ-treated glioma cells, the PG was pretreated for 6 h and then treated with TMZ for 48 h and then antimigration was analyzed. As shown in Figures 2(c) and 2(d), there were no significant statistic differences between PG-alone treatment and pretreated PG plus TMZ treatment suggesting the antimigration should be cotreated with PG and TMZ.

### 3.3. PG Enhanced the TMZ Inhibition of MMP Activities in U87MG Cells

**Figure 3:**
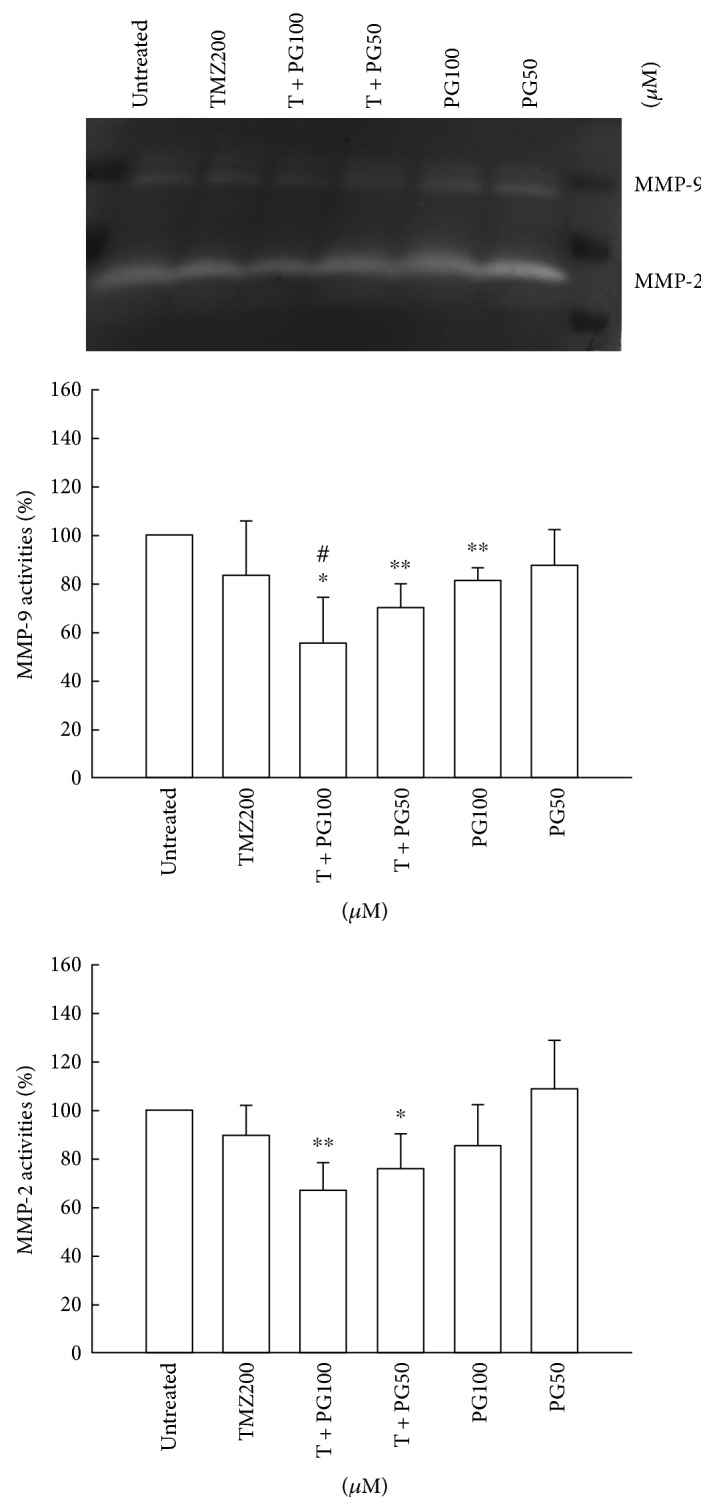
Analysis of MMP-2 and MMP-9 activities with TMZ, PG, or TMZ/PG treatment. U87MG cells (2 × 10^6^) were plated in 100 mm cultured dishes for 24 h and then treated with TMZ (200 *μ*M), PG (50 or 100 *μ*M), or their combination for 48 h. After treatment, the cultured media were collected and then centrifuged at 12,000 ×g for 10 min at 4°C to obtain supernatants. Aliquot protein (50 *μ*g) was used to evaluate the activities of MMP-2 and MMP-9 by zymography. These experiments were performed at least three times; a representative experiment is presented. Data indicate the densitometric values of various treated groups normalized to their corresponding untreated group. Significant differences for the untreated group and TMZ group were ^∗^*P* < 0.05, ^∗∗^*P* < 0.01, and ^#^*P* < 0.05.

MMP-9 and MMP-2 are important matrix metalloproteinases in the migration of glioma cells. MMP-9 and MMP-2 degrade the extracellular matrix and result in the migration of glioma cells to other normal tissue area. To determine whether PG has a potential role in the inhibition of MMP-9 or MMP-2 activity in U87MG cells either PG alone or PG/TMZ combination, we used zymographic analysis. As shown in Figure 3, the MMP-9 and MMP-2 activities were significant inhibition in PG/TMZ combination as compared with untreated cells. It is interesting to note that PG (100 *μ*M)/TMZ (200 *μ*M) combination resulted in a significant inhibition on MMP-9 activity as compared with TMZ (200 *μ*M) alone treatment. PG (50 *μ*M or 100 *μ*M) alone did not inhibit the activity of MMP-2.

### 3.4. PG Decreased ROS Production in TMZ Treatment

**Figure 4:**
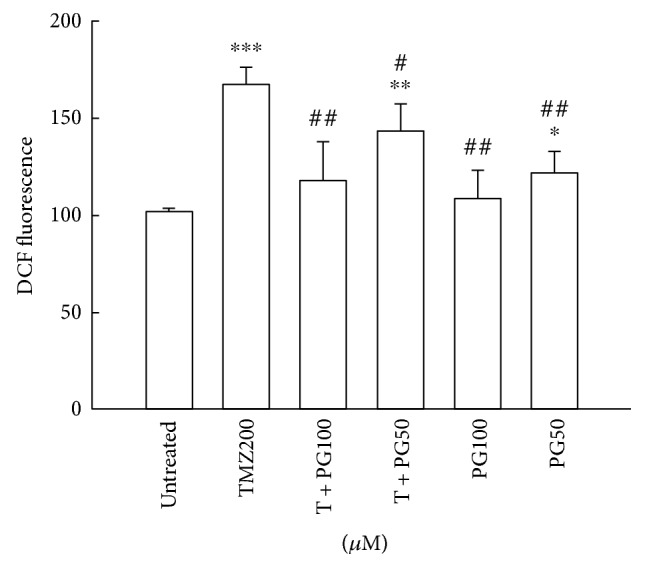
Evaluation of intracellular ROS with TMZ, PG, or TMZ/PG treatment. U87MG cells (1 × 10^6^) were plated in 60 mm cultured dishes for 24 h and then treated with TMZ (200 *μ*M), PG (50 or 100 *μ*M), or their combination for 48 h. After treatment, the cells were stained with 2′,7′-dichlorfluorescein-diacetate (DCFDA) for ROS analysis and were then evaluated by flow cytometry. Data represent the mean fluorescence intensity within the cells. The values are presented as mean ± standard deviation (*n* = 5–8) of individual experiments. Significant differences for the untreated group and TMZ group were ^∗^*P* < 0.05, ^∗∗^*P* < 0.01, ^∗∗∗^*P* < 0.001, ^#^*P* < 0.05, and ^##^*P* < 0.01.

To fully evaluate the possible involvement of ROS in the antimigration effects of PG/TMZ-combined treatment, we detected ROS accumulation in the treated cells. Increased ROS accumulation occurred in the TMZ-alone group (Figure 4). Treatment with PG (50 *μ*M or 100 *μ*M) alone had an inhibitory effect on ROS accumulation compared to that of TMZ alone. However, ROS inhibition was little enhanced by combined treatment with PG (50 *μ*M or 100 *μ*M) and TMZ. These results indicate that ROS generation may partially obstruct the antimigration effect of TMZ, and the inhibition of ROS by PG may be a critical event contributing to the antimigration effect of TMZ treatment.

### 3.5. PG/TMZ Combination Suppressed NF-*κ*B Activity

**Figure 5:**
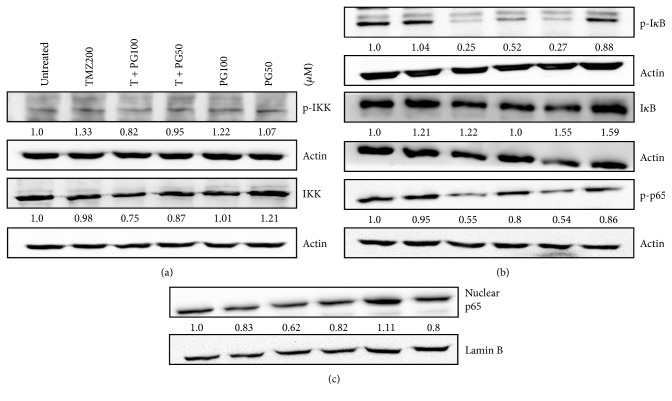
Expressions of (a) p-IKK and IKK; (b) p-I*κ*B, I*κ*B, and p-p65; (c) nuclear p65 with TMZ, PG, or TMZ/PG treatment. U-87 MG cells (2 × 10^6^) were plated in 100 mm cultured dishes for 24 h and then treated with TMZ (200 *μ*M), PG (50 or 100 *μ*M), or their combination for 48 h. After treatment, total proteins or nuclear proteins were extracted to assess various protein expressions. Fifty micrograms of protein were loaded onto a 12% SDS-polyacrylamide gel and evaluated by Western blotting. These experiments were performed at least three times; a representative experiment is presented. Data indicate the densitometric values of various treated groups normalized to their corresponding untreated group.

NF-*κ*B is known to regulate the expression of a number of MMPs. We therefore investigated the effect of PG and TMZ on the NF-*κ*B pathway in U87MG cells. The expression of NF-*κ*B pathway-related proteins such as p-IKK, p-I*κ*B, and p-p65 was examined by Western blot analysis. PG alone or in combination was quite effective in suppressing the constitutive activation of the NF-*κ*B pathway, including p-IKK, p-I*κ*B, and p-p65 in U87MG cells, but TMZ treatment alone had no marked effect on the constitutive NF-*κ*B activation pathway in U87MG cells (Figure 5).

### 3.6. Inhibition of Critical ROS-Generated Sites Enhanced the Antimigration Effect of TMZ on U87MG Glioma Cells

**Figure 6:**
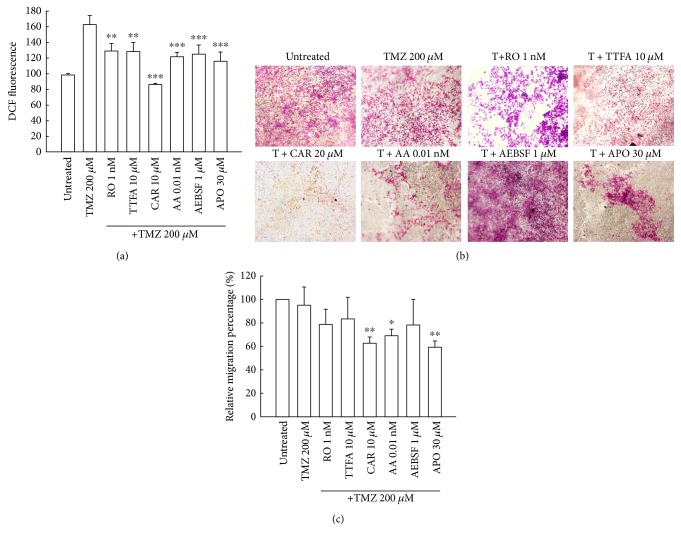
Inhibition of critical ROS events enhances antimigration with TMZ treatment. U-87 MG cells (1 × 10^6^) were plated in 60 mm cultured dishes for 24 h and then treated with TMZ (200 *μ*M) alone for 48 h or pretreated with 1 nM rotenone (RO, a complex I inhibitor), 10 *μ*M 2-thenoyltrifluoroacetone (TTFA, a complex II inhibitor), 10 *μ*M carboxin (CAR, a complex II inhibitor), 0.01 nM antimycin A (AA, a complex III inhibitor), 1 *μ*M 4-(2-Aminoethyl) benzenesulfonyl fluoride hydrochloride (AEBSF, a NADPH oxidase inhibitor), and 30 *μ*M apocynin (APO, a NADPH oxidase inhibitor) for 1 h, followed by TMZ (200 *μ*M) for 48 h. After treatment, the cells were stained with 2′,7′-dichlorfluorescein-diacetate (DCFDA) for ROS analysis and were then evaluated by flow cytometry. Data represent the fluorescence intensity within the cells. The values are presented as mean ± standard deviation (*n* = 5–8) of individual experiments. Significant differences for the TMZ group were ^∗∗^*P* < 0.01 and ^∗∗∗^*P* < 0.001. (b) U87MG cells (1 × 10^4^) were plated in 24-well Millicell hanging cell culture inserts in an 8 *μ*m polyethylene terephthalate membrane for 24 h and then treated with TMZ (200 *μ*M) alone for 48 h or pretreated with 1 nM rotenone (RO, a complex I inhibitor), 10 *μ*M 2-thenoyltrifluoroacetone (TTFA, a complex II inhibitor), 10 *μ*M carboxin (CAR, a complex II inhibitor), 0.01 nM antimycin A (AA, a complex III inhibitor), 1 *μ*M 4-(2-Aminoethyl) benzenesulfonyl fluoride hydrochloride (AEBSF, a NADPH oxidase inhibitor), and 30 *μ*M apocynin (APO, a NADPH oxidase inhibitor) for 1 h, followed by TMZ (200 *μ*M) for 48 h. Transwell migration assay was carried out. The migrated cells were stained using crystal violet. Random fields from each of the triplicate migration assays were counted using phase contrast microscopy (magnification 200x). (c) The dye was eluted with 33% acetic acid, and crystal violet absorbance was measured at 570 nm using a microplate reader. The values are presented as mean ± standard deviation (*n* = 5–8) of individual experiments. Significant differences for the TMZ group were ^∗^*P* < 0.05 and ^∗∗^*P* < 0.01.

The antioxidant effect of PG enhanced the antimigration effect of TMZ treatment. We further evaluated the critical ROS-generated sites in TMZ-treated U87MG cells. Various specific ROS-generated inhibitors were pretreated for 1 h and then treated with TMZ for 48 h. Finally, the cells were evaluated for intracellular ROS levels. All ROS-generated inhibitors reduced TMZ-induced ROS production (Figure 6(a)). The inhibition of migration with TMZ treatment was enhanced in the presence of carboxin (a complex II inhibitor), antimycin A (a complex III inhibitor), or apocynin (a NADPH oxidase inhibitor), but not in the presence of rotenone (a complex I inhibitor) (Figures 6(b) and 6(c)). These results indicated that ROS production induced by TMZ treatment occurs through mitochondrial respiratory chain complex I, complex II, complex III, and NADPH oxidase. Inhibition of ROS generated from complex II and III, and NADPH oxidase can enhance the antimigration effect of TMZ on U87MG cells.

### 3.7. PG Did Not Induce Cytotoxicity in the Normal Cell Lines

**Figure 7:**
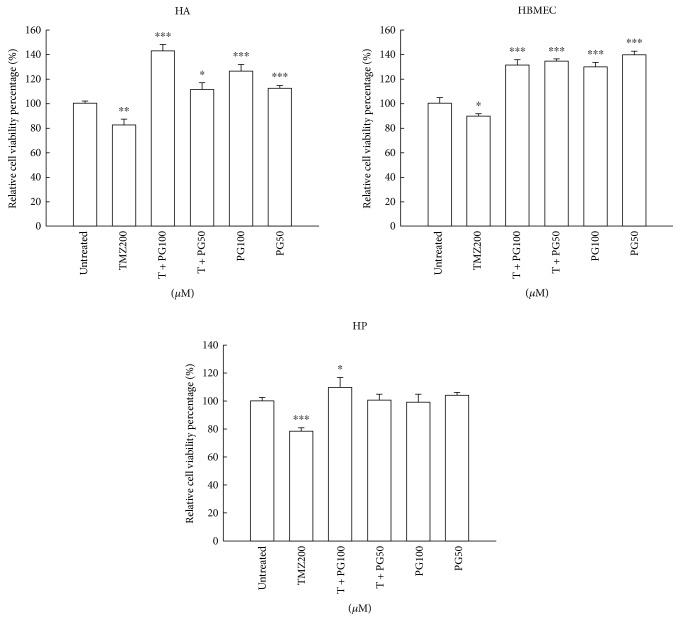
Evaluation of cell viability with TMZ, PG, or TMZ/PG treatment in normal cell lines. HA, HBMEC, and HP cells (1 × 10^4^) were seeded in each well of a 96-well cultured plate for 24 h and then treated with TMZ (200 *μ*M), PG (50 or 100 *μ*M), or their combination for 48 h. After treatment, cell viability was determined by an XTT-based assay. The values are represented as mean ± standard deviation (*n* = 5–8) of individual experiments. Significant differences for the untreated group were ^∗^*P* < 0.05, ^∗∗^*P* < 0.01, and ^∗∗∗^*P* < 0.001.

To evaluate whether 50 or 100 *μ*M PG induced toxic in normal neuron and glia cells, three human normal cell lines, HBVP (human brain vascular pericytes), HA (human astrocytes), HBMEC (human brain microvascular endothelial cells), and XTT analysis were used. As shown in Figure 7, TMZ treatment resulted in 80–90% cell viability in three normal cell lines. However, treatment of 50 or 100 *μ*M PG did not induce cytotoxicity in the three cell lines suggesting PG may protect normal cells during TMZ treatment.

## 4. Discussion

A critical step in cancer invasion is breaking through the ECM and invading the neighboring stroma. Control of the invasive nature of GBM cells may offer hope for more efficacious local therapy and improve the patient's quality of life [18]. Neutral proteases can alter capillary permeability by attacking ECM around the blood vessels. MMP-2 and MMP-9 destroy ECM and help breast and prostate cancer cells migrate to new places [19, 20]. In glioma cells, MMP-2 and MMP-9 are highly expressed and are involved in GBM migration and invasion [21]. Rojiani et al. reported that enhanced vascular proliferation, particularly at the brain-tumor interface, increased the capability of tumors to grow and invade in brain malignancy, possibly modulated by MMP-2 [22].

ROS regulation of cell migration and adhesion in endothelial-derived cells is an important mechanism [23], and it also provides a common trigger for many downstream pathways that directly mediate BBB oxidative damage [24]. The role of ROS in tumor metastasis involves complicated processes, including MET, migration, invasion by tumor cells, and angiogenesis around the tumor lesion [25, 26]. ROS generation may be induced intracellularly in either a NADPH oxidase-dependent manner or a mitochondria-dependent manner, by growth factors and cytokines (such as TGF beta and HGF) or by tumor promoters (such as TPA) capable of triggering cell adhesion, MET, and migration [27–29]. ROS has been found to directly regulate the expression and activity of MMPs in human endothelial cells [30], and several reports indicated that ROS was involved in the abnormal activation of these MMPs in several types of cancer cells, including glioblastoma [31]. Our results revealed that PG inhibited the expression of MMP-2 and MMP-9 and exerted an antimigration effect on TMZ-treated U87MG cells. TMZ induced an increased in ROS production by about 1.6-fold, and PG (100 *μ*M) significantly inhibited the ROS level in TMZ treatment. This seems to suggest that downregulation of the ROS level with TMZ treatment can enhance the inhibition of migration.

The ROS-dependent intracellular pathway activation relationship with tumor invasion includes the regulation of NF-*κ*B transcription factor located upstream of MMPs [30]. The elevation of the NF-*κ*B level in cancer may be the result of either exposure to proinflammatory stimuli in the tumor microenvironment or upregulation of the signaling pathway by upstream regulators [32]. In addition, the NF-*κ*B pathway is activated in glioblastoma-initiating cells undergoing differentiation, and blockade of this activation promoted the senescence of differentiating cells [33]. Appropriate control of NF-*κ*B activity, which can be achieved by gene modification or pharmacological strategies, would provide a potential approach to the management of NF-*κ*B-related tumors, including glioblastoma.

The NF-*κ*B-induced expression of MMPs has been found to be regulated by ROS generated from mitochondria and NADPH oxidase [34]. PG also possessed anti-inflammatory activity via downregulation of the NF-*κ*B pathway [35], but its potential effect on the NF-*κ*B signaling cascade in glioma cells has never been evaluated. Our previous study found that PG could reduce the proliferation and augment the chemosensitivity of a THP-1 leukemia cell line via extrinsic and intrinsic apoptotic pathways [14]. In this study, we noticed that the effect of PG/TMZ-combined treatment may be mediated by inhibition of the NF-*κ*B pathway, such as p-IKK, p-I*κ*B, and p-p65, in U87MG glioma cells. We also found that both p-I*κ*B and nuclear p65 in U87MG glioma cells were more suppressed by combined PG and TMZ. This indicates that the inhibition of the NF-*κ*B pathway enhanced by PG is a critical mechanism of antimigration in TMZ treatment. Furthermore, blockade of NADPH oxidase by diphenyleneiodonium abolishes NF-*κ*B activation and inhibits MMP-9 expression, indicating the role of ROS in NF-*κ*B-induced MMP expression [35]. In agreement with our results, the inhibitors of mitochondrial complex III (carboxin and antimycin A) and NADPH oxidase (apocynin) significantly inhibited the ROS level and migration with TMZ treatment, further indicating that inhibition of the ROS-mediated NF-*κ*B pathway in TMZ treatment is an important antimigration mechanism in glioma cells.

Many polyphenol compounds have been found to pass the blood-brain barrier, including epigallocatechin-3-gallate [36, 37]. PG is a polyphenol compound which contains hydrophobic propyl group and phenol group, and the compound structure of PG is smaller than epigallocatechin-3-gallate. However, Wu et al. demonstrated that the mean in vivo recoveries of both polyphenol compounds (+)-catechin and (−)-epicatechin in microdialysis probes were 38.3% and 29.1% in the brain [37]. We speculate that 50 or 100 *μ*M of PG may partially pass through the blood-brain barrier. The correct concentration of PG in the brain must be further detected. In our previous study demonstrated that U87MG cells express high oxidative stress status as compared with that of other glioma cells [38]. Our results show that TMZ induced ROS generation in glioma by 1.6-fold of untreated cells. The increased ROS generation in TMZ-treated U87MG cell may not further induce the migration activity.

Wu et al. demonstrated that a strong linear correlation between the cell migration rate and the amount of intracellular ROS suggests that ROS is an intermediate product to enhance cell migration [39]. Furthermore, an antioxidant *α*-tocopherol can decrease the migration rate by quenching the ROS production [39]. Our results showing that PG could block ROS generation induced by TMZ treatment suggested that ROS blocking by PG may provide partially antimigration effect. Accumulating reports indicate the transcription factor NF-*κ*B as a positive mediator of tumor metastasis, and the NF-*κ*B signaling pathway provides critical roles in neuroblastoma migration and invasion [40]. Our results demonstrating that PG inhibited the NF-*κ*B pathway resulted in MMP activity inhibition suggest that NF-*κ*B pathway inhibition may be the main mechanism on antimigration in PG/TMZ combination. Morgan and Liu indicate that certain NF-*κ*B-regulated genes play a major role in regulating the amount of ROS in the cell and ROS would modulate an NF-*κ*B response and that NF-*κ*B target genes would attenuate ROS to promote survival [41]. Our results found that PG/TMZ combination resulted in NF-*κ*B pathway inhibition but did not increase ROS generation suggesting those two phenomena (block ROS generation and NF-*κ*B pathway inhibition) may be independent events.

**Figure 8:**
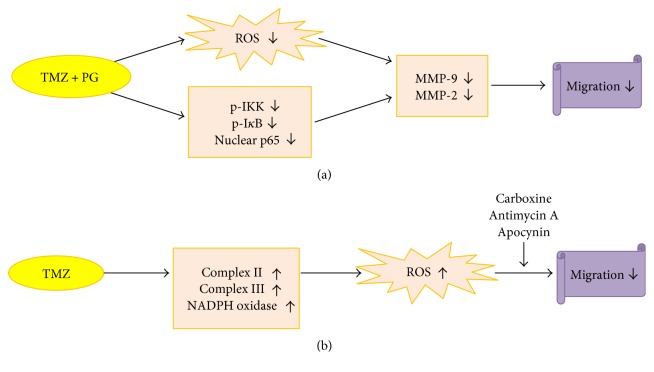
Diagrammatic scheme denoting the cascade of events in antimigration. (a) PG enhances the effect of TMZ on antimigration through inhibition of ROS generation, the NF-*κ*B pathway, and MMP activities; (b) inhibition of mitochondrial complex II, III, and NADPH oxidase contributes to the antimigration effect of TMZ.

In conclusion, our findings provide further scientific evidence that (1) PG enhances the antimigration effect of TMZ through inhibition of ROS generation, the NF-*κ*B pathway, and MMP activities (Figure 8(a)); and (2) TMZ induces ROS generation from mitochondrial complex I, II, and III and NADPH oxidase, and inhibition of complex III and NADPH oxidase enhances the antimigration effect of TMZ (Figure 8(b)).

## References

[1] Omuro A., DeAngelis L. M. (2013). Glioblastoma and other malignant gliomas: a clinical review. *The Journal of the American Medical Association*.

[2] Huang B., Zhang H., Gu L. (2017). Advances in immunotherapy for glioblastoma multiforme. *Journal of Immunology Research*.

[3] Zarnett O. J., Sahgal A., Gosio J. (2015). Treatment of elderly patients with glioblastoma: a systematic evidence-based analysis. *JAMA Neurology*.

[4] Caspani E. M., Crossley P. H., Redondo-Garcia C., Martinez S. (2014). Glioblastoma: a pathogenic crosstalk between tumor cells and pericytes. *PLoS One*.

[5] Narita Y. (2015). Bevacizumab for glioblastoma. *Therapeutics and Clinical Risk Management*.

[6] Fu P., He Y. S., Huang Q. (2016). Bevacizumab treatment for newly diagnosed glioblastoma: systematic review and meta-analysis of clinical trials. *Molecular and Clinical Oncology*.

[7] Lena A., Rechichi M., Salvetti A. (2009). Drugs targeting the mitochondrial pore act as cytotoxic and cytostatic agents in temozolomide-resistant glioma cells. *Journal of Translational Medicine*.

[8] Bache M., Bernhardt S., Passin S. (2014). Betulinic acid derivatives NVX-207 and B10 for treatment of glioblastoma--an in vitro study of cytotoxicity and radiosensitization. *International Journal of Molecular Sciences*.

[9] Vartanian A., Singh S. K., Agnihotri S. (2014). GBM’s multifaceted landscape: highlighting regional and microenvironmental heterogeneity. *Neuro-Oncology*.

[10] Tasaki T., Fujita M., Okuda T. (2016). MET expressed in glioma stem cells is a potent therapeutic target for glioblastoma multiforme. *Anticancer Research*.

[11] Chen C. H., Chang Y. J., Ku M. S., Chung K. T., Yang J. T. (2011). Enhancement of temozolomide-induced apoptosis by valproic acid in human glioma cell lines through redox regulation. *Journal of Molecular Medicine*.

[12] Hamishehkar H., Khani S., Kashanian S., Ezzati Nazhad Dolatabadi J., Eskandani M. (2014). Geno- and cytotoxicity of propyl gallate food additive. *Drug and Chemical Toxicology*.

[13] Jo E. J., Park S. J., Kim B. C. (2016). Propyl gallate sensitizes human lung cancer cells to cisplatin-induced apoptosis by targeting heme oxygenase-1 for TRC8-mediated degradation. *European Journal of Pharmacology*.

[14] Chen C. H., Lin W. C., Kuo C. N., Lu F. J. (2011). Role of redox signaling regulation in propyl gallate-induced apoptosis of human leukemia cells. *Food and Chemical Toxicology*.

[15] You B. R., Park W. H. (2011). The enhancement of propyl gallate-induced apoptosis in HeLa cells by a proteasome inhibitor MG132. *Oncology Reports*.

[16] Zheng J. M., Chen X. C., Lin M. (2011). Mechanism of the reduction of cerebral ischemic-reperfusion injury through inhibiting the activity of NF-kappaB by propyl gallate. *Yao Xue Xue Bao*.

[17] Taparia S. S., Khanna A. (2016). Procyanidin-rich extract of natural cocoa powder causes ROS mediated caspase-3 dependent apoptosis and reduction of pro-MMP-2 in epithelial ovarian carcinoma cell lines. *Biomedicine & Pharmacotherapy*.

[18] Toussaint L. G., Nilson A. E., Goble J. M. (2012). Galectin-1, a gene preferentially expressed at the tumor margin, promotes glioblastoma cell invasion. *Molecular Cancer*.

[19] Gil M., Kim Y. K., Kim K. E., Kim W., Park C. S., Lee K. J. (2016). Cellular prion protein regulates invasion and migration of breast cancer cells through MMP-9 activity. *Biochemical and Biophysical Research Communications*.

[20] Dayal S., Zhou J., Manivannan P. (2017). RNase L suppresses androgen receptor signaling, cell migration and matrix metalloproteinase activity in prostate cancer cells. *International Journal of Molecular Sciences*.

[21] Zhang F. Y., Hu Y., Que Z. Y. (2015). Shikonin inhibits the migration and invasion of human glioblastoma cells by targeting phosphorylated beta-catenin and phosphorylated PI3K/Akt: a potential mechanism for the anti-glioma efficacy of a traditional Chinese herbal medicine. *International Journal of Molecular Sciences*.

[22] Rojiani M. V., Alidina J., Esposito N., Rojiani A. M. (2010). Expression of MMP-2 correlates with increased angiogenesis in CNS metastasis of lung carcinoma. *International Journal of Clinical and Experimental Pathology*.

[23] Hurd T. R., DeGennaro M., Lehmann R. (2012). Redox regulation of cell migration and adhesion. *Trends in Cell Biology*.

[24] Pun P. B., Lu J., Moochhala S. (2009). Involvement of ROS in BBB dysfunction. *Free Radical Research*.

[25] Cichon M. A., Radisky D. C. (2014). ROS-induced epithelial-mesenchymal transition in mammary epithelial cells is mediated by NF-kB-dependent activation of snail. *Oncotarget*.

[26] Galadari S., Rahman A., Pallichankandy S., Thayyullathil F. (2017). Reactive oxygen species and cancer paradox: to promote or to suppress?. *Free Radical Biology & Medicine*.

[27] Panday A., Sahoo M. K., Osorio D., Batra S. (2015). NADPH oxidases: an overview from structure to innate immunity-associated pathologies. *Cellular & Molecular Immunology*.

[28] Simon H. U., Haj-Yehia A., Levi-Schaffer F. (2000). Role of reactive oxygen species (ROS) in apoptosis induction. *Apoptosis*.

[29] Rhyu D. Y., Yang Y., Ha H. (2005). Role of reactive oxygen species in TGF-beta1-induced mitogen-activated protein kinase activation and epithelial-mesenchymal transition in renal tubular epithelial cells. *Journal of American Society Nephrology*.

[30] Lian S., Xia Y., Khoi P. N. (2015). Cadmium induces matrix metalloproteinase-9 expression via ROS-dependent EGFR, NF-small ka, CyrillicB, and AP-1 pathways in human endothelial cells. *Toxicology*.

[31] Chiu W. T., Shen S. C., Chow J. M., Lin C. W., Shia L. T., Chen Y. C. (2010). Contribution of reactive oxygen species to migration/invasion of human glioblastoma cells U87 via ERK-dependent COX-2/PGE(2) activation. *Neurobiology of Disease*.

[32] Martins G. R., Gelaleti G. B., Moschetta M. G., Maschio-Signorini L. B., Zuccari D. A. (2016). Proinflammatory and anti-inflammatory cytokines mediated by NF-kappaB factor as prognostic markers in mammary tumors. *Mediators of Inflammation*.

[33] Xia W., Tian H., Cai X. (2016). Inhibition of SUMO-specific protease 1 induces apoptosis of astroglioma cells by regulating NF-kappaB/Akt pathways. *Gene*.

[34] Shinohara M., Adachi Y., Mitsushita J. (2010). Reactive oxygen generated by NADPH oxidase 1 (Nox1) contributes to cell invasion by regulating matrix metalloprotease-9 production and cell migration. *The Journal of Biological Chemistry*.

[35] Jung H. J., Kim S. J., Jeon W. K. (2011). Anti-inflammatory activity of n-propyl gallate through down-regulation of NF-kappaB and JNK pathways. *Inflammation*.

[36] Mähler A., Mandel S., Lorenz M. (2013). Epigallocatechin-3-gallate: a useful, effective and safe clinical approach for targeted prevention and individualized trearment of neurological diseases?. *The EPMA Journal*.

[37] Wu L., Zhang Q. L., Zhang X. Y. (2012). Pharmacokinetics and blood-brain barrier penetration of (+)-catechin and (-)-epicatechin in rats by microdialysis sampling coupled to high-performance liquid chromatography with chemiluminescence detection. *Journal of Agricultural and Food Chemistry*.

[38] Yang J. T., Li Z. L., Wu Y. J., Lu F. J., Chen C. H. (2014). An oxidative stress mechanism of shikonin in human glioma cells. *PLoS One*.

[39] Wu S. Y., Hou H. S., Sun Y. S., Cheng J. Y., Lo K. Y. (2015). Correlation between cell migration and reactive oxygen species under electric field stimulation. *Biomicrofluidics*.

[40] Zhi Y., Duan Y., Zhou X. (2014). NF-*κ*B signaling pathway confers neuroblastoma cells migration and invasion ability via the regulation of CXCR4. *Medical Science Monitor*.

[41] Morgan M. J., Liu Z. (2011). Crosstalk of reactive oxygen species and NF-*κ*B signaling. *Cell Research*.

